# Acinetobacter: A Rare Cause of Rapid Development of Cavitary Lung Lesion Following COVID-19 Infection

**DOI:** 10.7759/cureus.24366

**Published:** 2022-04-22

**Authors:** Tutul Chowdhury, Arjun Mainali, Amulya Bellamkonda, Nicole Gousy

**Affiliations:** 1 Internal Medicine, One Brooklyn Health System, Brooklyn, USA; 2 Medicine, American University of Antigua, New York, USA

**Keywords:** covid-19, ventilator-associated pneumonia, rare lung diseases, lung cavitation, multidrug-resistant acinetobacter

## Abstract

Cavitary lesions of the lungs are a very frequent picture found in clinical practices resulting from a wide range of pathological processes with variable duration of formation depending on infectious pathogens. Common organisms causing cavitary lesions are *Staphylococcus aureus*, *Klebsiella pneumoniae*, *Streptococcus pneumoniae*, *Haemophilus influenzae*, typical and atypical *Mycobacterium*, and *Aspergillus*. Herein, we are presenting a case that developed cavitary lesions in both lungs colonizing *Acinetobacter*, a rare causative agent, within less than two months of a positive coronavirus disease 2019 (COVID-19) infection.

## Introduction

The coronavirus disease 2019 (COVID-19) infection caused by the severe acute respiratory syndrome coronavirus 2 (SARS-CoV-2) and leading to severe acute respiratory syndrome (SARS) has been spreading rapidly worldwide with a deleterious effect on global health. Pulmonary lesions caused by virus infection will gradually be absorbed to complete dissipation, and very few severe patients may retain pulmonary interstitial inflammation and fibrosis. The repairing process of SARS-CoV-2-infected pulmonary lesions is not well known. For SARS patients in the later stage of recovery, pulmonary secondary/superinfections caused by bacteria, mold, and tuberculosis were very common, which sometimes lead to the formation of cavities and cystic lung changes in lungs, especially for severe patients who used antibiotics, glucocorticoids, and ventilator for a long time [1]. Causative bacteria are also variable across various studies; mostly gram-positive cocci such as *Staphylococcus aureus* and *Streptococcus pneumoniae*, and gram-negative bacteria such as *Haemophilus influenzae* are the common pathogens to cause early superinfection. Late superinfections are commonly caused by *Pseudomonas*, *Klebsiella pneumoniae*, *Escherichia coli*, *Enterobacter* spp., and rarely by *Acinetobacter* species [2]. Imaging features in bacterial superinfection can be variable and may often be difficult to diagnose in the presence of pre-existing COVID-19 pneumonia, especially when bilateral. Serial chest radiographs are helpful in the detection of new imaging findings [3]. Few studies explained new challenges in imaging pulmonary superinfections and co-infections in COVID-19 and explained how CT scans act as a useful modality to predict the likely organism since the routine bronchoscopy sampling techniques are limited in COVID-19 patients. Systematic assessment of the morphology of lung involvement on CT helps to narrow the differential diagnosis [2]. Herein, we report a case where the patient developed bilateral lung cavities post COVID-19 pneumonia due to late superinfection by a rare bacteria, *Acinetobacter baumannii*.

## Case presentation

A 58-year-old male with a past medical history of seizure disorder, cerebrovascular accident with right-sided weakness, heart failure with reduced ejection fraction (HFrEF) of 35-45%, hypertension, chronic hypoxic respiratory failure due to COVID-19 pneumonia post status tracheostomy on a ventilator, and a history of adrenal insufficiency was transferred from a nursing home after an episode of fever and hypotension in view of septic shock. His past medical history was significant for acute hypoxic respiratory failure due to COVID-19 pneumonia four months ago, followed by tracheostomy and percutaneous endoscopic gastrostomy (PEG) tube placement due to failed weaning trial. He also had methicillin-resistant *Staphylococcus aureus* (MRSA) bacteremia with right-sided empyema status post chest tube insertion during prolonged hospital stays, for which he was managed with a total of one month of antibiotic (vancomycin followed by ceftaroline).

On clinical examination, the patient was on tracheostomy on a ventilator maintaining saturation of 98-100% with a fraction of inspired oxygen (FiO2) of 40%. The patient was found to have tachycardia, and his blood pressure was on the lower side with a normal temperature. On chest examination, bilateral crepitation was noted. PEG tube stump on the right upper abdomen was clean. Healed heel ulcer was observed on the left foot and lower leg planter side. The patient was admitted to the medical floor for sepsis, likely from pneumonia, and was managed with a broad-spectrum antibiotic.

**Figure 1:**
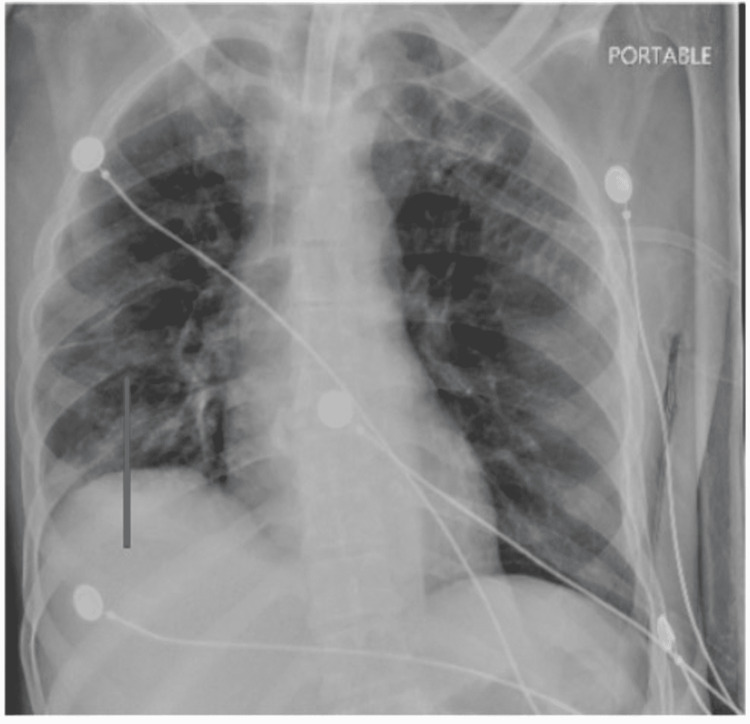
Chest X-ray showing patchy bilateral airspace disease, greatest in the right mid to lower lung. Taken during current patient admission.

**Figure 2:**
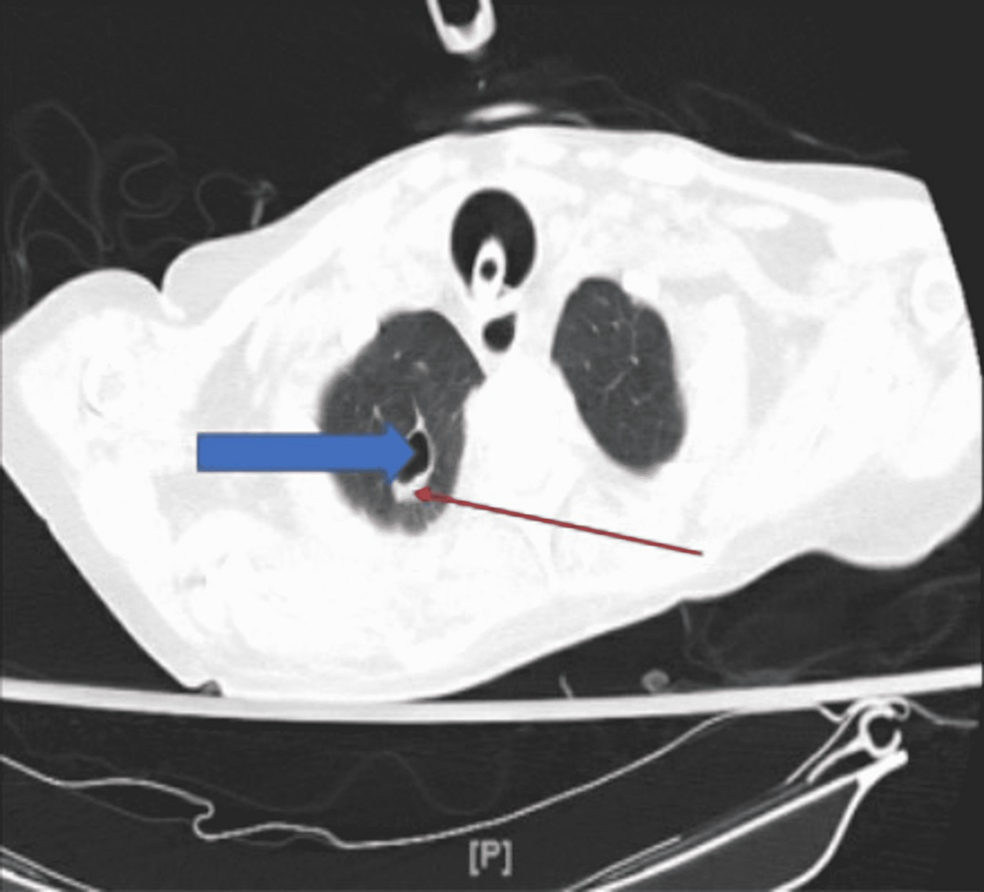
CT showing a thin-walled cavitary lesion in the apical segment of the right upper lobe. The red arrow is pointing to the thin walls of the cavitation, and the blue arrow is pointing to the cavitation as a whole.

**Figure 3:**
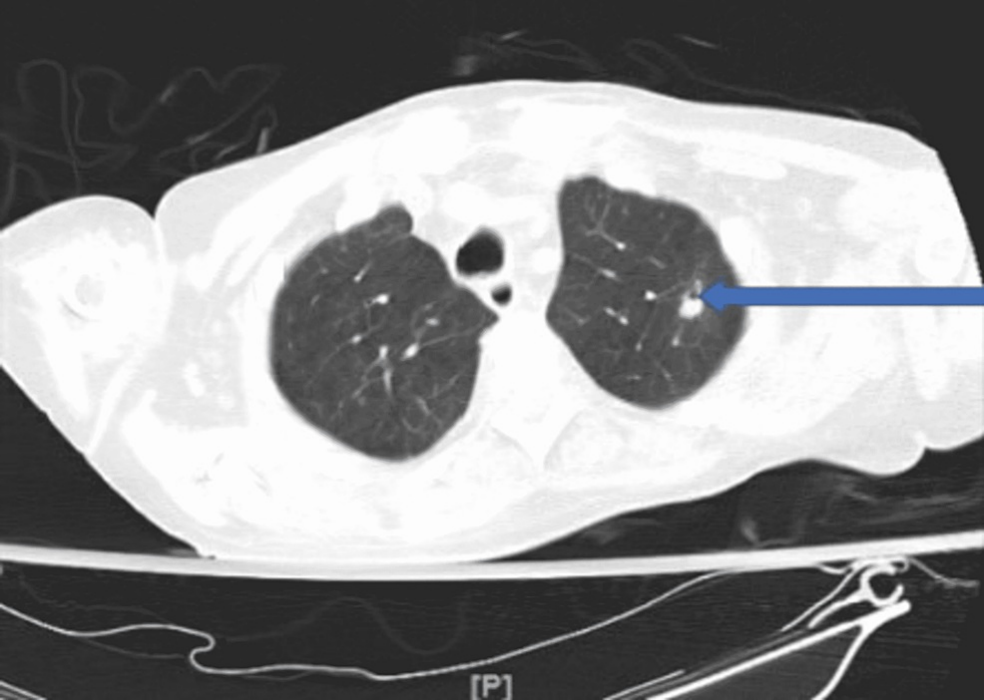
CT showing cavitary lesion in the apical posterior segment of the left upper lobe (blue arrow) taken during patient admission.

A complete blood count, metabolic panel, urine analysis, urine culture, blood culture, sputum gram stain and culture, COVID-19 test, imaging studies, and inflammatory markers were ordered during the time of admission. Significant lab findings were a high white blood cell count of 17.1 and an elevation of C-reactive protein (CRP) and erythrocyte sedimentation rate (ESR) at the time of admission. B-type natriuretic peptide (BNP) and troponin were negative. The blood culture was positive for *E. coli*; however, this was thought to be a false positive result as repeat blood cultures were negative. Sputum gram stain showed gram-negative rods, and culture was positive for *Acinetobacter baumannii*. Sputum for acid-fast bacilli was negative three times. Chest X-ray showed no significant changes (Figure 1). CT exhibited cavitary lesions in the apical segment of the right upper lobe with an air-fluid level, which was thin-walled (Figure 2), and the apical posterior segment of the left upper lobe (Figure 3). The patient was started on broader spectrum antibiotics including vancomycin, cefepime, and metronidazole, and was later switched to ceftazidime based on the sputum culture and sensitivity report and was instructed to complete the antibiotic duration based on the recommendation from the infectious disease team.

Previous CT of the chest two months prior to this admission was negative for any nodular and cavitary lesions. In terms of the previous hospitalization, the patient was treated for MRSA bacteremia three months prior to this admission.

## Discussion

Although CT scans can drastically differ between COVID-19 patients, common imaging findings include ground-glass opacities (GGOs), consolidative pulmonary opacities, interlobular septal thickening, and pleural thickening [4,5]. Of note, GGOs were seen in approximately 88% of confirmed COVID-19 cases, with 87.5% of those cases having bilateral lobe involvement, 76% having peripheral distribution, and 78.8% of these cases exhibiting GGOs in a multi-lobar distribution [4]. These findings have been shown to even precede the development of symptomatic COVID-19, or have prevailed in the scenario where a patient, despite having respiratory symptoms and a pathognomonic CT scan, presents with a false negative initially for the reverse transcription-polymerase chain reaction (RT-PCR) screening test [4]. Uncommon CT findings can also include pleural effusion, pericardial effusion, and a CT halo sign during the later stages of disease progression [4]. While there is a wide range of CT findings in those diagnosed with the COVID-19 virus, there is a noticeable absence of pulmonary cavitations being reported, especially in those who have long since recovered from the virus, such as our patient [4,5].

Cavitation is defined as an air-filled space developing within an area with established pulmonary consolidations, masses, or nodules. Cavitations form as a direct result of liquefactive necrosis within these lesions, which settle into the bronchial tree [6]. These lesions are typically a result of several bacterial, parasitic, fungal, or neoplastic pathogenesis [5,7]. To date, cavitations secondary to COVID-19 pneumonia have only been reported in the form of various case reports; however, there have been some retrospective reports describing the frequency of the complication [4,7]. One study reported that only 12 out of 689 (1.7%) patients admitted with COVID-19 pneumonia developed pulmonary cavitary lesions during the course of their disease [6]. However, there have been other reports indicating the frequency of cavitation development in as much as 11% of those with more severe cases of COVID-19 pneumonia [7].

Due to the rarity and novelty of the complication, its pathophysiology has not been entirely mapped out; however, many theories have been proposed [4,5,7]. Some have suggested that a combination of long-lasting mucous settling within the smaller bronchioles can perpetuate bronchial inflammation processes. Thus propagating the progression of fibrosis and the development of bronchiectasis, which eventually develop into cavitations [7]. This theory would explain why cavitations have developed in those with milder forms of COVID-19 [7].

Other hypotheses include the possibility that these cavitations are a result of co-infections with bacteria such as *Staphylococcus aureus* [2,7]. While this common infection is seen in many who develop these lesions, our patient's lesions were due to a co-infection with the rare *Acinetobacter* species. It is thought that the inflammation and subsequent bronchiectasis that can develop with COVID-19 pneumonia can create a breeding ground for bacteria, which can lead to cavitation development [7]. Additionally, there have been cases reported where cavitations did not exhibit any positive cultures, and those who did grow positive cultures were the minority of patients with this complication [8]. Rather, these cases suggest that there is a thrombotic effect of the COVID-19 virus that leads to the development of cavitations [7,8].

There are very few reported cases of *Acinetobacter* causing late lung cavitations, as seen in this patient, after recovering from COVID-19 pneumonia as of date [9]. The *Acinetobacter* species is a large but rare group of gram-negative bacteria that have the reputation of being multi-drug resistant [9]. Typically these bacteria will cause ventilator-associated pneumonia, especially in critically ill patients; recent studies have shown that approximately 1-28% of COVID-19 patients with the active disease on ventilators will subsequently develop a superimposed *Acinetobacter* infection while on the ventilator [9]. However, there are limited data regarding the pathogenesis of *Acinetobacter* in the development of new lung cavitations such as seen with this patient, especially in those where lung cavitations developed after the resolution of COVID-19 pneumonia.

Typical lung patterns on CT scans of those with COVID-19 pneumonia range wildly and differ between every patient. While GGOs are the pathognomonic findings associated with this viral infection, an important complication could be the development of lung cavitations, specifically those due to a superimposed bacterial infection. It is possible that the alveolar damage preceded by the virus can create a breeding ground for new bacterial infections to develop, thus close monitoring with frequent CT scans is recommended in monitoring disease progression [2,4,10].

## Conclusions

Until now, very few cases have been reported pointing to *Acinetobacter* as a causal agent for the acute development of lung cavities, especially in non-tuberculosis patients. Hence, its pathogenesis is so far unclear due to its rarity. Recognizing unusual etiologies is a crucial element in the diagnosis of disease, especially when there is so much yet to be learned about the SARS-CoV-2 virus and *Acinetobacter* species. Having this pathogen in the differentials may guide clinicians to diagnosis and choose treatment modalities early to prevent irreversible lung damage.

## References

[1] Chiang CH, Shih JF, Su WJ, Perng RP (2004). Eight-month prospective study of 14 patients with hospital-acquired severe acute respiratory syndrome. Mayo Clin Proc.

[2] Naranje P, Bhalla AS, Jana M, Garg M, Nair AD, Singh SK, Banday I (2021). Imaging of pulmonary superinfections and co-infections in COVID-19. [PREPRINT]. Curr Probl Diagn Radiol.

[3] Modi AR, Kovacs CS (2020). Hospital-acquired and ventilator-associated pneumonia: diagnosis, management, and prevention. Cleve Clin J Med.

[4] Salehi S, Abedi A, Balakrishnan S, Gholamrezanezhad A (2020). Coronavirus disease 2019 (COVID-19): a systematic review of imaging findings in 919 patients. AJR Am J Roentgenol.

[5] Selvaraj V, Dapaah-Afriyie K (2020). Lung cavitation due to COVID-19 pneumonia. BMJ Case Rep.

[6] Zoumot Z, Bonilla MF, Wahla AS (2021). Pulmonary cavitation: an under-recognized late complication of severe COVID-19 lung disease. BMC Pulm Med.

[7] Kurys-Denis E, Grzywa-Celińska A, Celiński R (2021). Lung cavitation as a consequence of coronavirus-19 pneumonia. Eur Rev Med Pharmacol Sci.

[8] Kruse JM, Zickler D, Lüdemann WM (2021). Evidence for a thromboembolic pathogenesis of lung cavitations in severely ill COVID-19 patients. Sci Rep.

[9] Rangel K, Chagas TPG, De-Simone SG (2021). Acinetobacter baumannii infections in times of COVID-19 pandemic. Pathogens.

[10] Ammar A, Drapé JL, Revel MP (2021). Lung cavitation in COVID-19 pneumonia. Diagn Interv Imaging.

